# Exosomal Cargo May Hold the Key to Improving Reproductive Outcomes in Dairy Cows

**DOI:** 10.3390/ijms22042024

**Published:** 2021-02-18

**Authors:** Natalie Turner, Pevindu Abeysinghe, Pawel Sadowski, Murray D. Mitchell

**Affiliations:** 1 Institute of Health and Biomedical Innovation—Centre for Children’s Health Research, School of Biomedical Sciences, Faculty of Health, Queensland University of Technology, Brisbane, QLD 4029, Australia; natalie.turner@hdr.qut.edu.au (N.T.); abeysinghe.abeysingh@hdr.qut.edu.au (P.A.); 2 Central Analytical Research Facility—Queensland University of Technology, Gardens Point, Brisbane, QLD 4000, Australia; pawel.sadowski@qut.edu.au

**Keywords:** exosome, mass-spectrometry, proteomics, SWATH, reproduction, fertility, dairy cow

## Abstract

The reproductive status of dairy cows remains a challenge for dairy farmers worldwide, with impaired fertility linked to a significant reduction in herd profitability, due in part to impaired immunity, increased metabolic pressure, and longer postpartum anestrous interval (PPAI). Exosomes are nanovesicles released from a variety of cell types and end up in circulation, and carry proteins, bioactive peptides, lipids, and nucleic acids specific to the place of origin. As such, their role in health and disease has been investigated in humans and animals. This review discusses research into exosomes in the context of reproduction in dairy herds and introduces recent advances in mass-spectrometry (MS) based proteomics that have a potential to advance quantitative profiling of exosomal protein cargo in a search for early biomarkers of cattle fertility.

## 1. Introduction

Dairy cow fertility has been in decline for the past 20 years [1,2,3]. Selective breeding for milk production traits, negative energy balance (NEB), poor health or infection during the transition period (3 weeks before and the 3 weeks after calving), and early pregnancy loss have all been attributed to this decline [3,4,5]. These factors are thought to be linked but the underlying biological mechanisms responsible for these perturbations to reproductive performance have not yet been fully established.

Although it is widely accepted that increased metabolic pressure due to increased milk production is associated with poor reproductive outcomes, average producing cows may also experience reproductive challenges [6]. There are reports that discuss the lesser significance of increased milk production on fertility, and instead highlight genetic potential, nutritional intake, health status and farm management as major contributing factors to fertility status of the cow [7]. However, reliable predictors of future reproductive performance remain to be determined.Body condition scoring (BCS), and more recently BCS linked to timing of pubertal onset, is one of the few key indicators used by dairy farmers to manage and predict herd profitability [8].

Heifers can be separated into high- and low-fertility groups based on their genetic merit and other measurable physical traits [9]. However, this model has been found to be substandard when trying to address underlying causes of subfertility, and newer models expressing the extremes of the fertility spectrum have been developed in order to better explore the mechanisms responsible for the decline in calving rates over the past two decades. Although these newer models have allowed for improved sampling and study of the physiological stresses leading to poor reproductive performance, the biological mechanisms driving the disease process resulting in subfertility remain to be elucidated.

Exosomes, nanovesicles of ≈30–150 nm in diameter, can be isolated from the bodily fluids of dairy cows (e.g., blood plasma, milk, and follicular fluid), and present a unique opportunity to studying the molecular cues that underlie poor reproductive performance [10]. Exosomes are most commonly formed by the inward budding of multivesicular bodies (MVB) in the cell and begin as intraluminal vesicles (ILVs), and play a critical role in cell–cell signaling [11,12]. The molecular contents of circulating exosomes derived from the blood plasma and milk of dairy cows have been characterized to some extent, and contain, for example, proteins, mRNA, micro(mi)RNAs, and lipids [10,13]. It is possible that miRNA contained in the blood plasma exosomes of dairy cows serve as an epigenetic regulator of biological signaling pathways, including inflammation, which in turn may affect reproduction and development of the fetus during pregnancy [14]. Additionally, qualitative differences in proteomic exosomal cargo have been previously established in milk and plasma samples between high- and low-fertility dairy cows, and between cattle with and without uterine infection [15,16,17]. Quantitative differences in exosomal proteins between these high- and low-fertility groups are yet to be fully elucidated and may hold the key to identifying potential biomarkers for fertility. Exosomes contained in the blood plasma, for instance, can provide a systemic snapshot of valuable information about the health-status of the animal, which may be directly or indirectly related to reproductive status. This review will focus on the potential application of exosome-derived biomarkers to predict and lead to improved bovine reproduction in relation to key aspects of dairy cow fertility.

## 2. Exosomes

### Formation and Function

**Figure 1:**
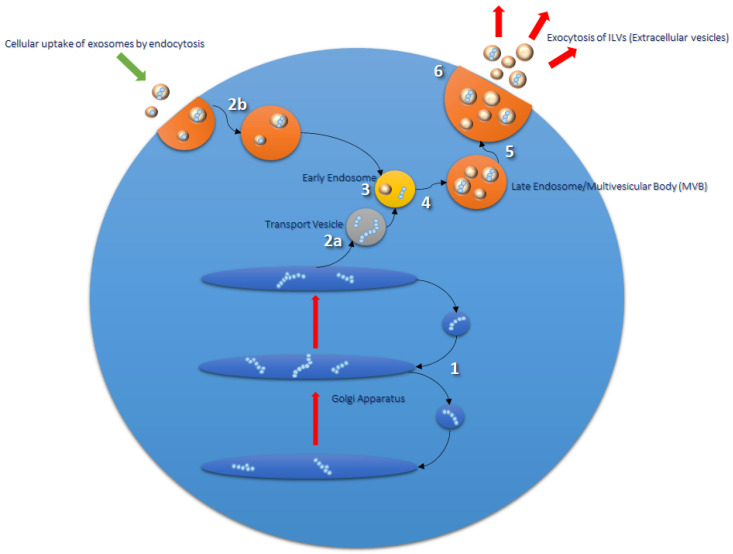
Routes of exosomal formation and release from the cell. The Golgi apparatus (**1**) transports and modifies proteins received from the endoplasmic reticulum (ER). Mature proteins and proproteins are transferred from the Golgi to endosomes via transport vesicles (**2a** and **3**). Early endosomes go on to form late endosomes/multivesicular bodies (MVBs) (**4** and **5**), which are composed of intraluminal vesicles (ILVs) formed from the inward budding of the endosomal membrane during the maturation process. Endosomal sorting complex required for transport (ESCRT) proteins are involved in this process and are found in ILV cargo. MVBs fuse with the plasma membrane of the cell to release their contents into the extracellular milieu; extracellular vesicles (EVs) (**6**). EVs are taken up by the cell via endocytosis or phagocytosis (**2b**) and transported to endosomal compartments and lysosomes for processing [37].

Within the cell there is a complex protein synthesis and sorting pathway, whereby protein folding and glycosylation begin in the endoplasmic reticulum (ER). Mature and proproteins are further modified as they pass through the Golgi apparatus, and following this are transported via transport vesicles to early endosomes (see Figure 1, next page) [18]. Early endosomes mature further into late endosomes, whereby they are transported to the cell surface and exocytosed via direct fusion with the plasma membrane [19]. Endocytosed materials may also be transferred to late endosomes and transported to lysosomes, or recycled back to the cell surface [20]. Late endosomes contain nucleic acids, proteins, lipids, and trans-Golgi Network (TGN)-derived transport vesicles; hence they are also termed multivesicular bodies (MVBs) [21]. ILVs within MVBs are released as extracellular vesicles (EVs), a subpopulation of which are termed exosomes [18,22]. Proteins involved in MVB formation and cargo sorting (endosomal sorting complexes required for transport (ESCRT) pathway) and its accessory proteins are also typically found in exosomes [22,23]. Therefore, ESCRT proteins such as Tumor Suppressor Gene 101 (TSG101) are used experimentally as positive exosomal markers, as are members of the tetraspanin family (CD9, CD63, CD81); the latter of which have recently been implicated as important mediators in mammalian reproduction [22,24,25].

Exosomal molecular cargo can be endocytosed by target cells via a number of different mechanisms; direct receptor–ligand interaction, through cell surface adhesion molecules such as integrins or cadherins that initiate endocytosis, or by the opsonization of exosomes inducing phagocytosis in the recipient cell [26,27]. It has been suggested that the uptake of exosomes may also depend on the recipient-cell type, as a study involving exosomes isolated from various cancer cell lines demonstrated differences in uptake by recipient cells regardless of the cell type of exosomal origin [28]. This suggests that exosomes can interact with any cell type, independent of the cell from which they themselves are derived, albeit by different mechanisms of endocytosis. Interestingly, Sung et al. (2020) confirmed pathfinding behaviour of cells as they migrate towards exosomal tracks in 2D and 3D models, and created a double reporter system to follow the release, uptake, and acidification of exosomal deposits in internalized compartments containing exosomes [29]. The results of these studies present promising directions for future research when considering the use of exosomes for targeted therapeutics.

Whereas exosomes were historically thought to contain cellular waste, more recent exosomal profiling has resulted in the understanding that they are intrinsic to cell maintenance, cell–cell signaling, immune modulation, and progression of tumor-derived cells and metastasis [22]. This has led to research into their ability to carry biomarkers of disease in easily attainable biological fluids such as blood, saliva, and urine [30,31,32,33], and their potential as therapeutic targets and delivery vehicles [26,30,34]. Currently, researchers have begun to establish EV profiles that will assist in determining the proportions of the various EV subtypes in any given biological sample, with the aim to better understand heterogenous populations of EVs and their distinct functions [35,36].

## 3. Bovine Reproduction

The reproductive health of dairy cows has been associated with a number of physiological factors and environmental factors. Heat stress has been implicated as an epigenetic modifier than may negatively impact upon the reproductive status of offspring [38,39], while NEB has been linked to poor transition around the time of calving and metabolic stress [40,41]. Importantly, non-esterified fatty acid (NEFA) surplus as a result of NEB has been shown to result in poor immune function and increased likelihood of uterine infection [40]. Inflammatory mediators from the prostaglandin (PG) family are known to play a part in reproductive processes in cattle, and as such have been the subject of investigations surrounding impaired fertility in dairy herds [42]. Qin and colleagues (2020) examined the effects of high NEFA concentrations on PG production in bovine endometrial (BEND) cells and observed decreased levels of prostaglandin E_2_ (PGE_2_) and prostaglandin F_2α_ (PGF_2α_) in cell culture media supernatant compared to controls [43]. Similarly, cows with metritis were found to have a differential abundance of common uterine bacteria compared with healthy cows [44]. Researchers have therefore attempted to establish ways to better manage cattle during times of physiological and metabolic challenge in hopes of improving reproductive health. For example, micronutrient supplementation during the transition period improved outcomes without altering the methylation state of the cows [45]. Thus, factors affecting reproductive performance of dairy herds are various and complex, and ways of determining intervention at an earlier stage may improve outcomes at a minimal cost to farmers and herds.

Exosomes have been the focus of bovine studies examining effects on implantation and embryo development. Two separate studies confirmed that exosomes derived from the bovine uterus increased gene and protein expression of the pregnancy-recognition-associated protein interferon-tau (IFN-τ) when cocultured with bovine embryos in vitro [46,47]. Another study implicated a role in exosome secretion from both conceptus and endometrium in facilitating crosstalk during the attachment period, while exosomes derived from follicular fluid have been shown to improve oocyte competence and resistance to environmental stressors such as heat shock [48,49]. Collectively, these studies suggest that exosomes are widely involved in bovine reproduction, thus supporting further evaluation of their contents and function.

While the protein cargo of exosomes has been somewhat characterized qualitatively, larger scale in-depth studies of quantitative differences between high- and low-fertility groups have not been conducted [13,17,50,51]. Dysregulation of the immune system, metabolic perturbations around the time of calving, and impaired embryonic-maternal crosstalk during implantation have all been associated with poor reproductive outcomes, and all of which exosomes are known to play a part [2,6,13,17,47,52]. Quantitative differences in exosomal protein cargo may have a significant impact on the overall health of dairy cows, upon which fertility may be directly or indirectly impacted. Differences may also serve as a valuable tool for predicting reproductive outcomes early on in the life of the cow and warrants further investigation.

### 3.1. The Immune System

**Figure 2:**
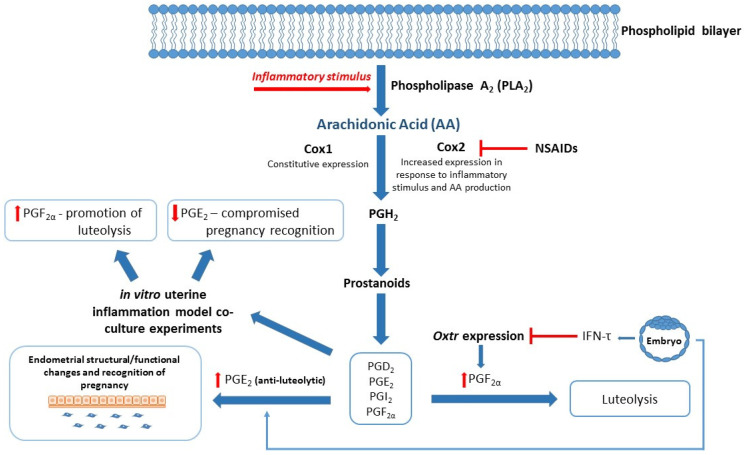
Blended model of reproduction and inflammation: Arachidonic Acid (AA)/Eicosanoid Pathway. Fatty acid cyclooxygenase 1/2 (*Cox 1/2*) converts AA to downstream effector molecules (Prostanoids and Prostaglandins (PGs)) following inflammatory stimuli. Interferon-tau (IFN-τ) produced by the conceptus inhibits Oxytocin receptor (*Oxtr*) expression and prevents luteolysis of luteinized granulosa cells to maintain progesterone secretion. IFN-τ stimulates PGE_2_ production in the endometrium, resulting in structural and functional changes required for pregnancy recognition. In vitro studies show altered expression of PGF_2α_ and PGE_2_ when exposed to inflammatory stimuli, which in turn may compromise events leading to successful establishment of pregnancy. Nonsteroidal anti-inflammatory drugs (NSAIDs) target the PG inflammatory cascade by inhibiting *Cox2* expression and reducing production of PGH_2_ and associated inflammatory mediators.

Successful reproduction in dairy cows relies on a competent immune system, especially during the periparturient period. Compromised immunity is associated with poor transition during the calving period and significant physiological stress, resulting in increased risk of postpartum uterine infection, mastitis, and an extended postpartum anestrous interval (PPAI). Studies have focused on various aspects of the immune system to better understand reproductive failings around early embryonic loss, postpartum uterine infection, and associated poor reproductive outcomes. Exosomes carry lipid mediators derived from arachidonic acid (AA), and enzymes involved in their synthesis, including inflammatory mediators associated with reproduction [53,54,55]. For example, PGs are small lipid compounds classed as eicosanoids, which among a diverse number of actions can behave as inflammatory mediators that are not only upregulated during infection and inflammation, but also play a critical role in establishment and maintenance of pregnancy in cattle [42,56,57]. PGE_2_ and PGF_2α_ are responsible for establishing or inhibiting bovine pregnancy, respectively [42]. Upregulation of inflammatory pathways during critical time points in the reproductive cycle of dairy cows could therefore have a severe impact on their reproductive health (see Figure 2). In an in vitro model of uterine inflammation, PGE_2_ and PGF_2α_ were found to be differentially expressed by bovine endometrial epithelial (bEEL) and stromal (bCSC) cells when exposed to inflammatory stimuli [58]. In further experiments, bEEL expression of PGF_2α_ was increased when coincubated with plasma exosomes derived from dairy cows with uterine infection [51]. Fatty acid cyclooxygenase-2 (COX2), which is upstream of the proinflammatory PGE_2_, has been highlighted as a potential target for therapies including the use of nonsteroidal anti-inflammatory drugs (NSAIDs) (see Figure 2) [2,40], although NSAIDS have previously been found to be ineffectual on Cox2 mRNA levels [59]. Interestingly, NSAIDS were successful in inhibiting lipopolysaccharide (LPS)-induced PGE_2_ and tumor necrosis factor-alpha (TNFα) mRNA production, indicating a mechanism of action separate to Cox2 activity [59]. A recent meta-analysis aimed to compare antibiotic with non-antibiotic methods (e.g., NSAIDs) of treatment for acute puerperal metritis (APM) in postpartum cattle [60]. Unfortunately, due to a shortage of comparable studies, the researchers were unable to perform the analysis for non-antibiotic methods, therefore the use of NSAIDs to treat postpartum uterine infection in cattle remains largely unverified.

### 3.2. The Transition Period

The transition period is a demanding phase in the life of dairy cows and challenging from the farm management perspective. It is typically defined as the period ranging from 3 weeks before and after calving [61] and represents a time of metabolic stress for the dairy cow, as the animal undergoes immense physiological changes in preparation for and during early lactation. Dairy cows that have been selectively bred for milk production traits experience greater metabolic pressure associated with increased milk production. Subsequently, this results in a greater incidence of postpartum uterine infection and mastitis, leading to ongoing health issues and negative implications for further reproduction [1,2,41,62]. Markers of metabolic distress such as β-hydroxybutyrate (BHB), triacylglycerols (TAG) and fatty acids (FA) were found to be altered in the blood plasma [2,8,61]. In addition to this, hypocalcemia resulting in ‘milk fever’ can occur, which results in the death of approximately 1 in 20 affected cows, reduces both the productive lifespan and milk production with each milk fever episode, and comes with associated costs of treatment and prevention [1,41,63]. The impact of metabolic distress during the transition period on future calving is of interest to reproductive studies. Increased metabolic pressure around the time of calving leads to lengthened PPAI and pre- and postovulatory dysfunction, which can significantly delay return to estrous and time to mating and is therefore of major concern to dairy farmers who operate under a seasonal-calving pasture-based system [2,8].

Numerous studies have focused on the link between BCS, NEB, and feed-intake during the transition period as a method of immunomodulation, in hopes of improving management of the transition dairy cow [64,65,66,67]. The use of exosomes as a potential source of biomarkers for low- versus high-risk populations of dairy cows has been investigated, with promising, although inconclusive, results [13]. Exosomes derived from the blood plasma of healthy versus dairy cows with cytological endometritis have been found to differ in protein composition when analyzed by liquid chromatography–mass spectrometry (LC-MS), which included proteins associated with innate immunity, acute immune response, and immune regulation [68]. Similarly, an in vitro study applied blood plasma exosomes isolated from dairy cows with and without uterine infection to endometrial cell lines to study their effects on PG production and found a decrease in luteolytic promoter PGF_2α_ produced by cells treated with exosomes derived from the infected cows [51]. This suggests the involvement of PGF_2α_ in disrupting normal reproductive processes and offers a potential target for improving outcomes in these animals. Despite this, the transition period still proves to be a challenging time for dairy farmers and their herds, and further research is required to better identify at-risk cows in hopes of preventing postpartum infection and maintaining reproductive efficiency.

Thus far, partly due to the ethical nature of conducting in vivo experiments, studies have steered towards in vitro modeling of bovine uterine infection. However, this may not be representative of the full spectrum of physiological mechanisms involved in, and leading to, high- or low-fertility and susceptibility to reproductive disruption in early life and during the transition or postpartum period. Bodily fluid samples obtained from cattle with and without disease may already be compromised regarding differences in molecular content, as it would be expected that inflammatory/disease markers would be present in affected animals at the time of disease occurrence. A more useful and predictive method of testing for differences would require sampling at the baseline stage, long before cattle experience reproductive and immune challenges. For example, sampling may occur around the time of puberty or earlier in order to establish a predictive model of reproductive performance and predisposition for disease in the early stages of reproductive life. Currently, Fertility Breeding Value (FBV) and BCS are the only tools available to dairy farmers to assist in the herd selection process, which does not consider the individual genetics or physiology of animals, but merely relies on physical attributes and genetic lineage as predictors [9,69]. Early biomarkers of fertility would aim to provide the dairy industry with reliable data that can assist in herd selection and lessen the burden of operational costs associated with poor reproductive performance. While lipid and inflammatory mediators transported by exosomes have been linked to reproduction in cattle, differences in protein cargo may give a better understanding of cattle fertility and the mechanisms that underlie perturbations to healthy reproduction.

## 4. Epigenetics of Reproduction

Epigenetic regulation of gene expression has been well studied with regards to mammalian development [70,71,72,73,74]. However, a new area of epigenetics is developing following research into the role of miRNAs as epigenetic modulators and has been reviewed recently [75,76]. Briefly, the epigenome is controlled at the base level by the expression of genes that encode for a group of enzymes, termed DNA methyltransferases (DNMTs) [71,73]. DNMTs catalyze the transfer of methyl groups to a specific part of DNA—CpG islands—as a way of altering gene expression [71]. miRNA performs modulatory actions at the epigenetic level by targeting DNMTs and histone deacetylases (HDACs) [75]. miRNA also has a direct impact on protein abundance via regulation at the translational level. Binding of miRNA to 3′ untranslated regions (UTRs) of target mRNA transcripts results in gene silencing or degradation, dependent on whether binding is imperfectly matched to the target sequence, or complimentary [77]. The epigenetic–miRNA regulatory loop also controls miRNA expression through DNA methylation, histone modification and RNA, and aberrations to these control mechanisms are associated with pathological health states [75,78,79]. Researchers have started to explore differential miRNA expression in hopes of finding early biomarkers of disease [80,81,82].

Bovine blood sera and exosomes have been subjected to miRNA profiling, and while 282 shared miRNAs were identified, 12 miRNAs were found to be differentially expressed between sera and exosomes [83]. Circulating miRNA has been shown to be a predictor of early pregnancy [84,85], and exosomal miRNA an indicator of early pregnancy loss in a cloned cattle study using somatic cell nuclear transfer (SCNT)-derived embryos [86]. The bovine estrous cycle, oocytes and preimplantation embryos have also been studied with regards to their specific exosomal and cell-free miRNA profiles. Subsequently, it was found that differential miRNA expression occurs during various stages of the estrous cycle and altered miRNA expression is associated with developmental competence of both oocytes and embryos [87,88,89,90]. Collectively, these results suggest that miRNA of exosomal and circulating origin may play an important role in regulating bovine reproduction. Correlative studies between miRNA and protein abundance would provide a comprehensive overview of the mechanisms behind systemic and local molecular regulation linked to reproductive outcomes.

## 5. Proteomics of Exosomes Derived from Bodily Fluids

### 5.1. Mass Spectrometry

Mass spectrometry (MS) is the technique of choice for determining the abundance of hundreds to thousands of proteins and continues to evolve through advancements in instrumentation, data acquisition modes and data analysis software. Its utility in protein analysis has a long history and has been extensively reviewed elsewhere [91,92,93,94]. In brief, methods for the effective formation of molecular ions from liquid or gas were established in the 1980s, and subsequently this led to the development of mass analyzers that were capable of determining the mass or structure of polypeptides with a high degree of sensitivity and accuracy [95,96,97,98]. MS systems are now commonly integrated and coupled with LC (LC-MS), which is the preferred method for analyzing samples with a high degree of complexity [98]. Initially widely used for peptide and protein identification in data-dependent acquisition (DDA) studies, MS instruments are now capable of peptide quantitation by labeled, relative, or targeted (absolute quantitation) methods, termed data-independent acquisition (DIA) [91,93,99,100].

In relation to dairy cow reproduction, MS has been utilized to perform thorough and reproducible analyses of bovine plasma, milk, follicular fluid, and uterine flushings [101,102,103]. To provide a better understanding of the signaling pathways associated with reproduction, exosomes isolated from milk of dairy cows have also been analyzed using a range of MS strategies in a number of studies [16,104,105,106]. Additionally, charge detection mass spectrometry (CDMS) and label-free spectral counting have been used successfully to characterize and quantify exosomes from milk and colostrum [104,105,107], and both milk and plasma exosomes have undergone qualitative analysis in DDA studies [4,16]. What is currently lacking in the field is a thorough quantitative analysis of the bovine blood plasma exosomal proteome, which may provide a better systemic snapshot of overall health and pathways associated with fertility, and thus clues to reproductive status in dairy cows. Table 1 summarizes what is currently known, and what remains to be established in relation to the role of exosomes of various origin and dairy cow reproduction.

DDA and targeted methods of MS, while effective, can be costly and/or only applicable to a limited number of samples. More recently, techniques such as sequential window acquisition of all theoretical mass spectra (SWATH-MS), termed next-generation proteomics, have emerged that allow the analysis of a greater number of samples with greater quantitative precision and impressive proteome coverage [116,117,118].

### 5.2. Next-Generation Proteomics

First described by Gillet et al. (2012), SWATH-MS is a variant of DIA that has already been applied to a large number of proteomic studies, including the analysis of exosomal protein cargo [116,119,120,121]. A major advantage of SWATH-MS approach is that quantitation is conducted using fragment ions, which are collected for all ionizable peptides in a sample, irrespective of their abundance. This is achieved using wide precursor isolation windows, which cover the expected mass range of all precursor ions. This effectively eliminates a bias in quantitation that other proteomics strategies have typically suffered from and permits a larger number of proteins across larger cohorts of samples to be analyzed with fewer missing values [117,118]. In its original implementation introduced by Gillet and colleagues, the highly complex nature of SWATH-MS data is dealt with using spectral libraries, however more recently, algorithms for library-free analysis have been developed [119,122,123].

Compared to other quantitative proteomics methods, the ease at which data is acquired is also a significant advantage, as once the precursor isolation scheme is set and method optimized for a particular sample type, analysis of different samples of the same type can be performed using the same method. A collaborative study looking at reproducibility and accuracy of SWATH-MS data detected and quantified >4000 proteins from Human embryonic kidney 293 (HEK293) cells in a 2-h run, and this was reproducible across multiple laboratories [124]. This allows proteomics studies to be performed on a much larger scale than originally feasible, with a high level of reproducibility and accuracy similar to that of targeted methods, but without the constraints of one-time data acquisition, as has been previously demonstrated [124,125]. The most promising feature of SWATH-MS in agriculture is that the data generated is ideal for retrospective quantitative analysis. SWATH-MS data may be exploited by remining them for new insights as genomic databases improve or as new compositional questions arise such as the ones derived from epigenetics analysis.

### 5.3. Current Challenges

Irrespective of MS approach employed, the analysis will largely depend on sample processing prior to MS. Highly abundant exosome proteins could compromise quantitation of low abundant cargo proteins of reproductive tissue origin and thus of biomarker potential. Enrichment of exosome populations of interest are therefore key to a successful outcome. The current methods of exosome purification involve sequential centrifugation, ultrafiltration, and size-exclusion chromatography, although there is currently no ‘gold-standard’ for exosome isolation [126,127,128,129,130]. These strategies, however, do not enrich for specific populations of exosomes that may be carrying the information specific to compromised fertility in cattle and further enrichment may be required [35]. This becomes even more critical when analyzing exosomes from bodily fluids and, in particular, from blood plasma, where the presence of several highly abundant plasma proteins such as albumin, globulins and fibrinogen may limit the overall number of exosome proteins detected in the study [131]. Furthermore, in the case of multistep enrichment, reproducibility of exosome preparation will have a significant impact on the ability of MS-based methods to reflect a true link between protein abundance and a biological phenomenon under study.

## 6. Conclusions

Suboptimal fertility in dairy cows has been attributed to acquired conditions such as poor uterine health, the adaptation to the transition period, and maternal-embryonic crosstalk in early pregnancy. Fertility status in dairy cows may also be determined at a much earlier timepoint due to factors stemming from genetic variants, which manifests in vivo as alterations to signaling pathways related to reproduction. Whether the fertility is a result of an acquired condition or inherited, the body responds in-kind by releasing exosomes that contain bioactive cargo that may provide a clue to cattle fertility [17,50,51]. Exosome research is a rapidly developing area of investigation for diagnostic and prognostic purposes. The qualitative and quantitative difference between exosomal cargo associated with different physiological conditions is determined using numerous ‘omics’ technologies and quantitative MS is at the forefront of this research. Specifically, a next-generation proteomics approach that relies on SWATH data acquisition to explore biomarkers of fertility on exosomes isolated and enriched from bovine blood plasma is currently being undertaken (unpublished data). Future research will aim to build on this concept through the study of miRNA on cellular function and signaling pathways related to fertility status of the animal, in hopes of developing targeted therapeutics to improve reproductive performance in cattle.

## Figures and Tables

**Table 1:** Summary of knowledge relating to exosomes and dairy cow reproduction.

Known	Not Known	Future Direction
Characterization of plasma exosomes derived from high- and low-fertility dairy cows [16].	‘Gold standard’ for exosome isolation is still a matter of contention.	Further optimization of exosomal isolation protocols specific to downstream application.
Characterization of bovine milk exosomes [16].	-	-
Established proteome profile of plasma exosomes derived from high- and low-fertility dairy cows [4,10].	Quantitative proteomic profile of exosomal cargo in circulating bovine exosomes.	SWATH-MS proteomic analysis of circulating exosomes in high- and low-fertility dairy cows to confirm quantitative differences and identify biomarker candidates related to good/poor reproductive outcomes.
Established proteome profile of bovine exosomes derived from milk, follicular fluid and uterine flushings [47,48,105,107,108].	Comprehensive quantitative proteomic profile of exosomes derived from bovine milk, follicular fluid and uterine flushings.	SWATH-MS proteomic analyses of exosomes derived from these biological fluid types to obtain a more complete understanding of the connection between physiological processes involved in dairy cow reproduction.
Characterization of bovine endometrial inflammation via in vitro inflammatory model utilizing bovine endometrial epithelial (bEEL) and stromal cells (bCSC) [58]. Exosomes derived from cows with uterine infection were found to decrease PGF_2α_ production in bEEL, but not bCSC cell lines [51]. Exosomes derived from cows at high- or low-risk of metabolic dysfunction differentially regulate eicosanoid gene expression in bEEL and bCSC cell lines [50].	In vitro studies utilizing novel protein biomarkers associated with healthy/aberrant reproduction.	Pathway analysis of potential biomarkers identified in protein studies and ongoing in vitro experiments to confirm biological function/impact of candidate biomarkers on eicosanoid gene and protein expression.
Exosome-derived uterine miRNAs from dairy cows are involved in blastocyst development and regulation of cytokines and chemokines [109,110].	Effect of miRNA knockdown on the function in relation to regulation of reproductive processes.	miRNA knockdown/knockout studies to confirm involvement of miRNA on the regulation of bovine reproductive processes.
Established miRNA profiles of bovine plasma- and milk-derived exosomes [111,112,113].	Comparative studies relating to exosomal miRNA profiles of high- and low-fertility dairy cattle.	Perform qualitative and quantitative analysis of exosomal miRNA in high- and low-fertility groups.
Immune challenges are associated with poor reproductive outcomes in dairy cows [41,62,114,115].	Relationship between immune status and poor reproductive outcomes needs further clarification.	Continuing studies on inflammatory mediators and their relationship to reproductive processes.

## Data Availability

Not applicable.

## Abbreviations

AA	Arachidonic acid
APM	Acute puerperal metritis
BCS	Body condition scoring
bCSC	Bovine stromal cells
bEEL	Bovine endometrial epithelial cells
BHB	β-hydroxybutyrate
CDMS	Charge detection mass spectrometry
COX1/*Cox1*	Fatty acid cyclooxygenase-1
COX2/*Cox2*	Fatty acid cyclooxygenase-2
NEB	Negative energy balance
DDA	Data-dependent acquisition
DIA	Data-independent acquisition
DNMT	DNA methyltransferase
ER	Endoplasmic reticulum
ESCRT	Endosomal sorting complexes required for transport
EV	Extracellular vesicles
FA	Fatty acids
FBV	Fertility breeding value
HDAC	histone deacetylase
HEK293	Human embryonic kidney 293
ILV	Intraluminal vesicle
IFN-τ	Interferon-tau
LC	Liquid chromatography
LPS	Lipopolysaccharide
miRNA	microRNA
MS	Mass spectrometry
MVB	Multivesicular bodies
NEB	Negative energy balance
NEFA	Nonesterified fatty acid
NSAID	Nonsteroidal anti-inflammatory drug
PG	Prostaglandin
PGE_2_	Prostaglandin E_2_
PGF_2α_	Prostaglandin F_2α_
PPAI	Postpartum anestrous interval
SCNT	Somatic cell nuclear transfer
SWATH-MS	Sequential window acquisition of all theoretical mass spectra
TAG	Triacylglycerols
TGN	*trans*-Golgi network
TNFα	Tumor necrosis factor-alpha
TSG101	Tumor Suppressor Gene 101
UTR	Untranslated region
